# Interspecific Hybridization and Mitochondrial Introgression in Invasive *Carcinus* Shore Crabs

**DOI:** 10.1371/journal.pone.0017828

**Published:** 2011-03-14

**Authors:** John A. Darling

**Affiliations:** Molecular Ecology Research Branch, National Exposure Research Laboratory, United States Environmental Protection Agency, Cincinnati, Ohio, United States of America; Ecole Normale Supérieure de Lyon, France

## Abstract

Interspecific hybridization plays an important role in facilitating adaptive evolutionary change. More specifically, recent studies have demonstrated that hybridization may dramatically influence the establishment, spread, and impact of invasive populations. In Japan, previous genetic evidence for the presence of two non-native congeners, the European green crab *Carcinus maenas* and the Mediterranean green crab *C. aestuarii*, has raised questions regarding the possibility of hybridization between these sister species. Here I present analysis based on both nuclear microsatellites and the mitochondrial cytochrome C oxidase subunit I (COI) gene which unambiguously argues for a hybrid origin of Japanese *Carcinus*. Despite the presence of mitochondrial lineages derived from both *C. maenas* and *C. aestuarii*, the Japanese population is panmictic at nuclear loci and has achieved cytonuclear equilibrium throughout the sampled range in Japan. Furthermore, analysis of admixture at nuclear loci indicates dramatic introgression of the *C. maenas* mitochondrial genome into a predominantly *C. aestuarii* nuclear background. These patterns, along with inferences drawn from the observational record, argue for a hybridization event pre-dating the arrival of *Carcinus* in Japan. The clarification of both invasion history and evolutionary history afforded by genetic analysis provides information that may be critically important to future studies aimed at assessing risks posed by invasive *Carcinus* populations to Japan and the surrounding region.

## Introduction

The anthropogenic introduction of populations into novel ecological contexts allows exploration of a wide range of phenomena that may influence evolutionary diversification [1], [2], [3]. Hybridization is one such mechanism that not only plays an important general role in shaping evolutionary trajectories, but also factors strongly in the establishment, spread and ecological impact of biological invasions [4], [5]. The capacity of hybridization to facilitate adaptive evolution and speciation has now been widely recognized [6], [7]. However, introgression of genomic elements across species boundaries also has the potential to disrupt genetic complexes generated through divergent adaptive evolution; the observation of interspecific hybridization between introduced and native species has thus led to concerns regarding the genetic integrity and evolutionary viability of native taxa, particularly those threatened by other stressors [8], [9], [10], [11]. More generally, both inter- and intraspecific hybridization may result in the formation of novel genetic types with potential for increased invasiveness relative to parental populations [2], [12], [13], [14]. Numerous empirical examples now exist of interspecific hybridization leading to the emergence of populations with novel invasive characteristics [6], [7], [15], and there is growing evidence that intraspecific admixture can result in increased genetic and phenotypic variance among introduced populations, with corresponding increases in the potential for rapid adaptation in recipient environments [16], [17], [18].

The rising frequency with which anthropogenic dispersal brings together previously allopatric lineages provides numerous opportunities to examine genetic exchange associated with recent hybridization events. To date the bulk of such research has focused on interspecific hybridization between native and invasive species. The vast majority of this literature has addressed plant taxa [13], with studies of interspecific hybridization involving invasive animal populations being principally limited to fish [5]. At the same time, efforts aimed at understanding the genetics of hybridizing invasive species have rarely considered hybridization events between multiple invasive taxa [19]. Although a number of important recent studies have explored intraspecific admixture following multiple independent introductions of animal taxa [16], [20], there are few cases that describe interspecific hybridization between multiple introduced species [21], [22]. Such studies may be important not only for their contribution to understanding hybridization dynamics, but also for clarifying the taxonomic identity of invasive populations. Cryptic hybridization can obscure both invasion history and, potentially, ecological distinctions that may prove relevant to the assessment of risks associated with biological invasions [23]. A number of recent studies have emphasized the value of genetic analysis in uncovering cryptic evolutionary diversification potentially relevant to invasion risk [24], [25], [26], and unrecognized hybrid lineages may represent an important component of this cryptic genetic diversity.

Shore crabs of the genus *Carcinus* provide a promising opportunity to examine interspecific genetic exchange between invasive animal species. While the European green crab *Carcinus maenas* Linnaeus has achieved a cosmopolitan distribution through anthropogenic dispersal, with established populations on all non-polar continents, its sole congener *Carcinus aestuarii* Nardo has been introduced to a more limited geographic range [27], [28], [29], [30]. In their native ranges the two species occupy largely non-overlapping distributions, with *C. maenas* found along the Atlantic coast of Europe and Africa from northern Scandinavia to Mauritania and *C. aestuarii* limited to the Mediterranean, although there has been some speculation as to the possible existence of a transition zone in southwestern Iberia [28], [31]. Genetic analyses based on mitochondrial loci indicate that the two species are well defined [32], a finding consistent with observations of diagnostic morphological criteria that reliably distinguish the species [33].

In Japan, crabs described as *C. aestuarii* were first reported in Tokyo Bay in 1984 and had spread as far southwest as Dokai and Sagami Bays by the 1990s [28]. The existence of *C. maenas* in Japan was suggested only later, when genetic analysis revealed the presence of mitochondrial haplotypes from both sister species [30]. It is notable that *C. maenas* has still generally not been recognized as a distinct presence in Japanese populations. Morphological analysis of Japanese *Carcinus* has largely supported the view that these crabs belong to *C. aestuarii* [33] and more recent ecological studies in Tokyo Bay recognize the population there as *C. aestuarii* [34]. The observation of several male crabs identified as *C. aestuarii* but possessing carapace width to length ratios characteristic of *C. maenas* provides the only morphological indication that some crabs may derive from mixed parentage [33]. Nonetheless, genetic analyses generally support a hypothesis of mixed species origin for Japanese *Carcinus*. Based on mitochondrial DNA haplotypes, Geller *et al.* [30] suggested that both *C. aestuarii* and *C. maenas* had independently invaded Japan. In contrast, Bagley and Geller [27] later used limited nuclear microsatellite data to infer that the Japanese population arose as the consequence of a single introduction from a native source population possessing both *C. maenas* and *C. aestuarii* mitochondrial haplotypes. This argument was supported primarily by the observation of low microsatellite diversity in Japan and the inference that multiple introductions from both Atlantic Europe and the Mediterranean would likely have conferred a much more diverse founding population. Both studies noted the possibility of hybrid origins for Japanese populations, but the absence of direct comparisons of nuclear and mitochondrial datasets precluded direct tests of that hypothesis. More recently, Darling *et al.* [29] explicitly argued for hybrid origins of the Japanese invasion based on combined analysis of mitochondrial COI and nuclear microsatellite data. These studies have led to some circumspection regarding the identity of Japanese *Carcinus* in recent assessments of invasion risk in that region [35].

This study extends on previous work by comprehensively addressing the hypothesis of a hybrid origin for invasive *Carcinus* population in Japan. Both mitochondrial COI sequence data and multilocus genotypes based on nine nuclear microsatellite loci were generated for 159 crabs collected from Tokyo and Dokai Bays. Analysis of these data confirms hybrid origin for the Japanese *Carcinus* population, and suggests that hybridization has resulted in massive introgression of a *C. maenas* mitochondrial haplotype into a predominantly *C. aestuarii* nuclear background. In light of the observed patterns of genetic variation in Japan, it seems most likely that the Japanese invasion derives from a single introduction from a hybrid population in the native range. This conclusion, along with genetic characterization of this previously undescribed hybrid lineage, may have important ramifications for understanding the invasion risks posed by Japanese *Carcinus* populations.

## Results

### Phylogenetic analysis

**Figure 1:**
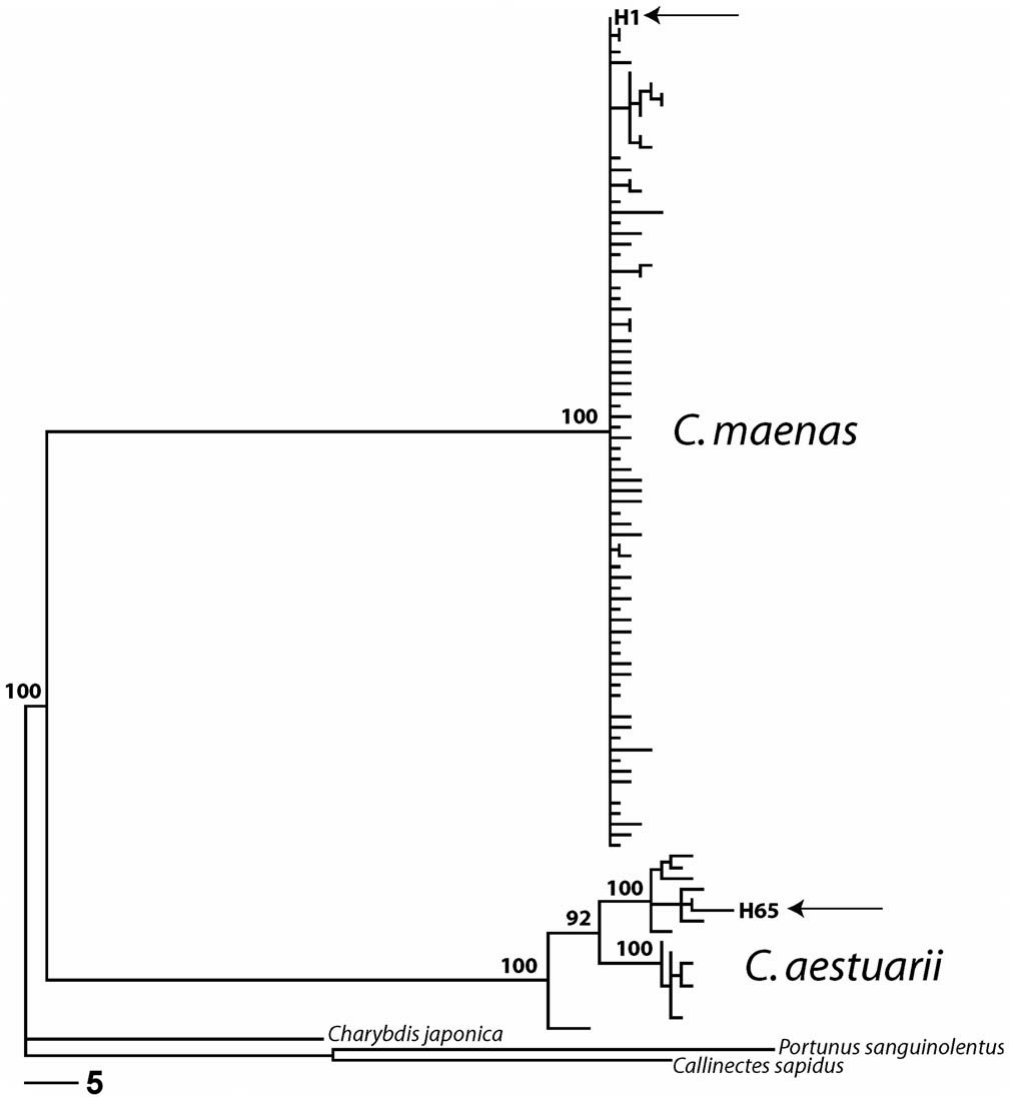
Phylogenetic tree determined by Bayesian inference. Posterior probabilities are indicated for major clades with support over 90%. Arrows indicate the two haplotypes present in Japan, H1 and H65. Scale bar represents 5 expected substitutions per site.

Bayesian inference of phylogenetic relationships based on the mitochondrial COI gene clearly indicates the divergence between the two sister species *C. maenas* and *C. aestuarii*, consistent with previous studies (Figure 1). Monophyly of the genus *Carcinus* relative to three portunid outgroups is strongly supported, and 100% posterior probability is given to both species lineages, as well as to two independent lineages within *C. aestuarii*. The only two COI haplotypes observed in Japan, H1 and H65, are assigned unambiguously to *C. maenas* and *C. aestuarii*, respectively. Mean Kimura 2-parameter genetic distances between the two species was 10.6% (0.08% within *C. maenas*, 2.5% within *C. aestuarii*) and the distance between invasive haplotypes H1 and H65 was 10.1%. H1 previously has been recognized as the single most common *C. maenas* haplotype in both the native and invasive ranges of that species; H65, in contrast, has been reported only from Japan (Darling et al. 2008). However, H65 does belong to a strongly supported clade (100% posterior probability) comprising haplotypes derived entirely from the eastern Mediterranean (Naples, Italy) and not observed in a more western population (Banyuls-sur-Mer, France).

### Population genetic structure within Japan

**Figure 2:**
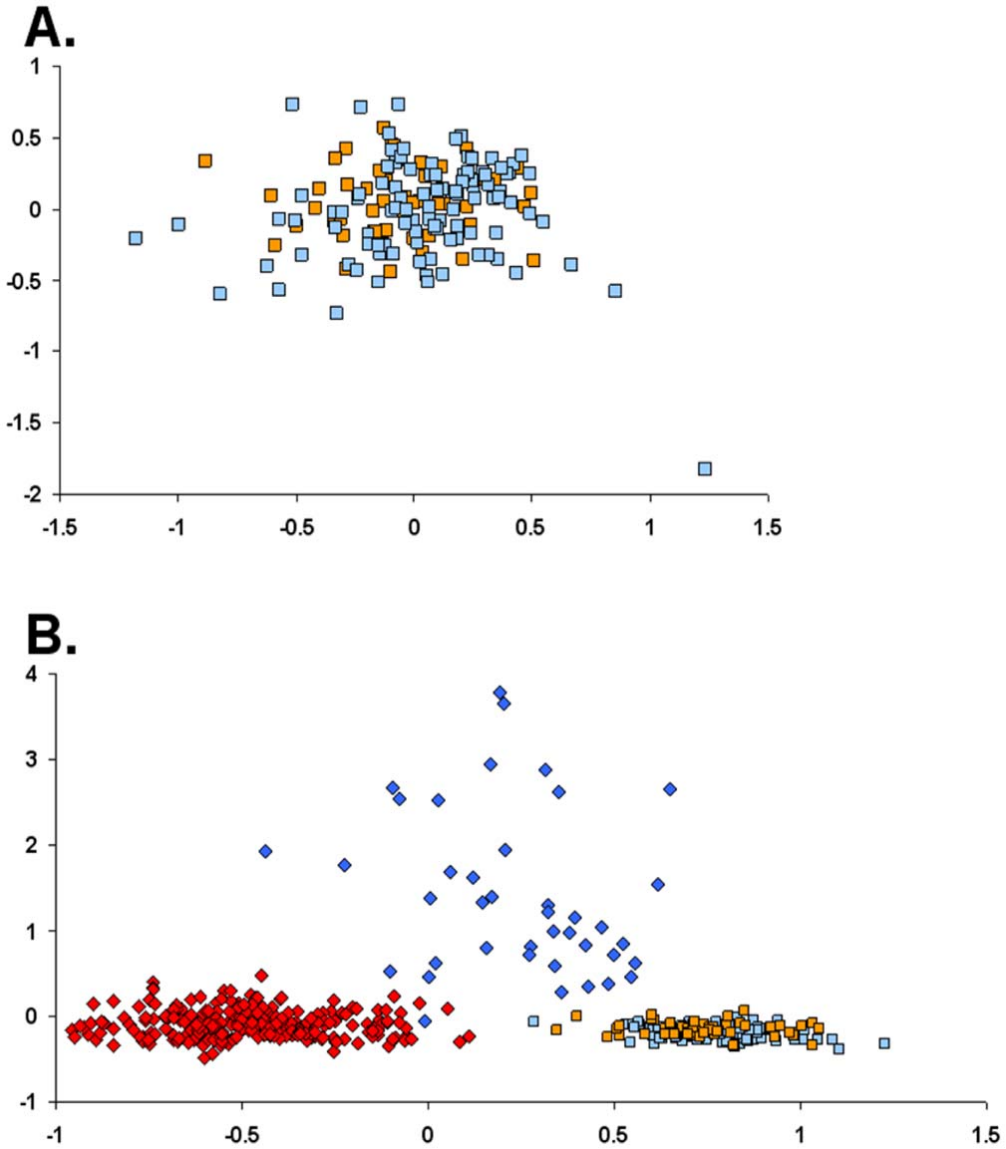
Factorial correspondence analysis of microsatellite genotype data. A) analysis of Japanese individuals alone; factor 1 (x axis) accounts for 6.49% of genetic variance, factor 2 (y axis) accounts for 6.18%. B) analysis of native and Japanese populations; factor 1 (x axis) accounts for 2.99% of genetic variance, factor 2 (y axis) accounts for 1.94%. Orange squares, Japanese individuals with COI haplotype H1; light blue squares, Japanese individuals with COI haplotype H65; red diamonds, native *C. maenas*; blue diamonds, native *C. aestuarii*.

**Figure 3:**
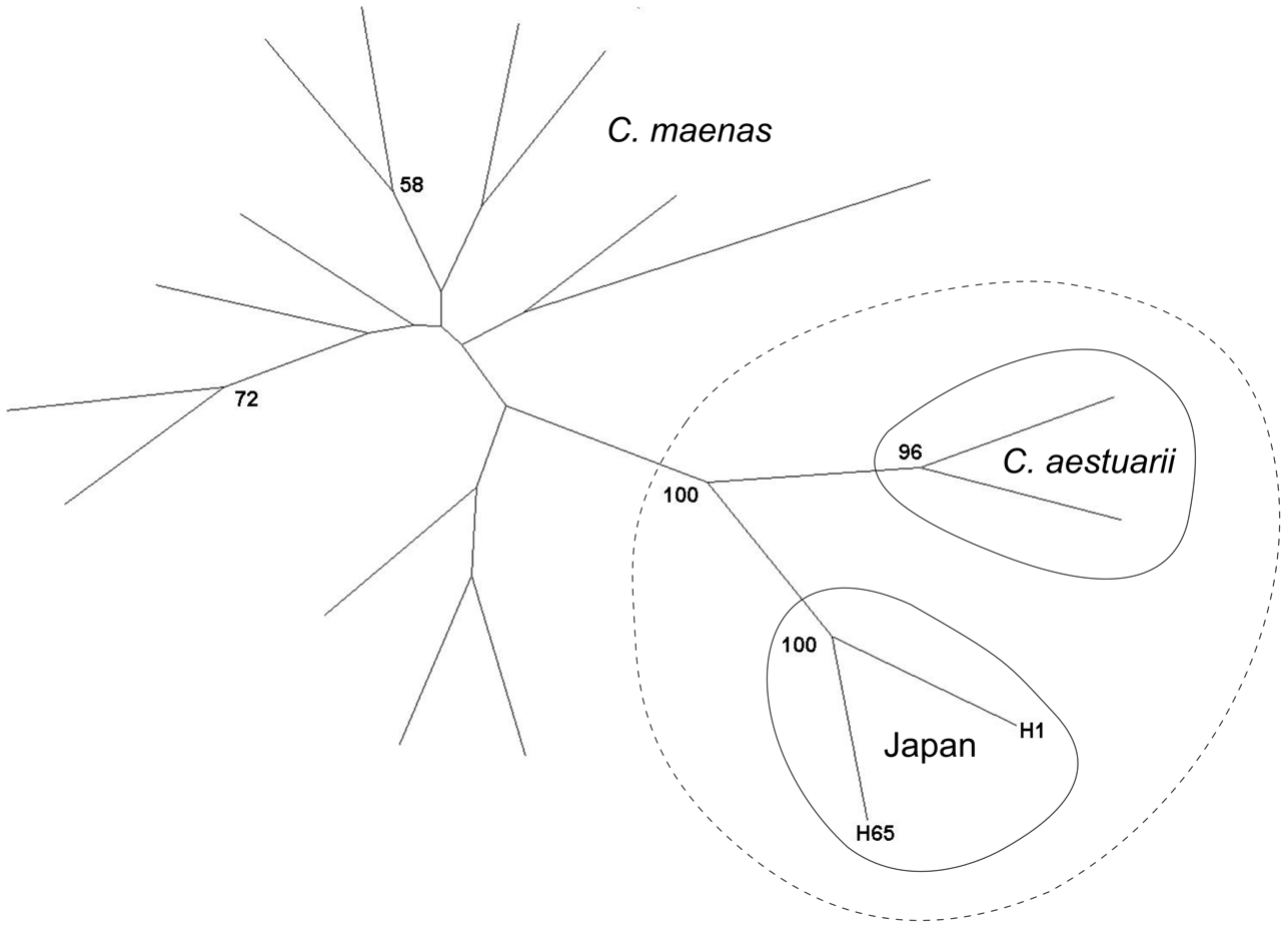
Neighbor joining analysis based on microsatellite chord distance. Japanese crabs were divided into groups harboring *C. maenas* COI haplotype H1 or *C. aestuarii* haplotype H65; all other branches represent collection sites from a previously published global dataset [29]. The dashed line indicates a strongly supported group comprising both *C. aestuarii* and Japanese crabs. Bootstrap values (1000 replicates) are shown only for those nodes with greater than 50% support.

Mitochondrial haplotypes were distributed unevenly among Japanese populations (Table 1), with H1 appearing significantly more frequently in Dokai Bay than in Tokyo Bay (*P*<0.0001, Fisher's exact test). In contrast, AMOVA based on nuclear microsatellite loci revealed no overall genetic differentiation between populations in the two Bays (Table 2, differentiation by site), although pairwise analysis did indicate marginally significant differentiation between Dokai Bay and one Tokyo Bay site (Table 3). More strikingly, AMOVA revealed no genetic differentiation at microsatellite loci between Japanese individuals possessing H1 and those possessing H65 (Table 2, differentiation by haplotype). This result was supported by FCA, which revealed a single cluster of Japanese genotypes regardless of associated mitochondrial haplotype (Figure 2A). This cluster was distinct from clusters defined by native *C. maenas* and native *C. aestuarii* (Figure 2B). The failure of microsatellite loci to distinguish between Japanese individuals with *C. maenas* and *C. aestuarii* COI haplotypes is reflected in complete cytonuclear linkage equilibrium, both within individual sampling sites and within the Japanese population as a whole (Table 4). Neighbor joining analysis based on microsatellite chord distances reveal a cluster of Japanese crabs harboring both H1 and H65 mitochondrial haplotypes with 100% bootstrap support (Figure 3). This Japanese cluster diverged from a similarly well-supported cluster comprising native *C. aestuarii*; however, both Japanese crabs and native *C. aestuarii* were grouped together to the exclusion of all native *C. maenas* populations with 100% support. Measures of both gene diversity and allelic richness were lower in Dokai Bay than in Tokyo Bay, although these differences were not significant (Table 5). Allele frequency distributions indicate that of 37 alleles present across all loci in Tokyo Bay, 14 (nearly 38%) have been lost in Dokai Bay. In contrast, no alleles were observed in Dokai Bay that were not also observed in Tokyo Bay.

**Table 1:** Summary of *Carcinus* collections.

Collection site	*n*	H1	H65	proportion H1
Tokyo Bay site 1 (TB)	50	12	38	0.2400
Tokyo University of Fisheries (TUF)	13	8	5	0.6154
Shinhama-ko lagoon (SK)	63	13	50	0.2063
Shinhama Bay (SHI)	15	2	13	0.1333
Tokyo Bay overall	141	35	106	0.2482
Dokai Bay (DOK)	18	16	2	0.8888
**total**	**159**	**51**	**108**	**0.3208**

Frequencies of mitochondrial COI haplotypes at four locations within Tokyo Bay and one location within Dokai Bay. H1 corresponds to *C. maenas* and H65 to *C. aestuarii* (see Figure 1).

**Table 2:** Analysis of Molecular Variance.

	*By site*			*By haplotype*		
	Variance components	Percentage of variation	Fixation index	Variance components	Percentage of variation	Fixation index
Among populations	−0.00332	−0.31	−0.00311(*P* = 0.68328)	−0.00728	−0.68	−0.00682(*P* = 0.97556)
Within populations	1.07412	100.31	-	1.07434	100.68	-

**Table 3:** Pairwise population differentiation among sampling sites (*F*_ST_).

	DOK	TB	TUF	SK	SHI
DOK	-	*0.52051*	***0.04297***	*0.98926*	*0.15039*
TB	−0.00268	-	*0.16211*	*0.27734*	*0.76758*
TUF	**0.03318**	0.01073	-	*0.17480*	*0.98047*
SK	−0.01515	0.00223	0.00765	-	0.99902
SHI	0.01554	−0.01060	−0.03674	−0.02814	-

*F*_ST_ values are shown below the diagonal, associated P values are shown in italics above the diagonal. Significant differentiation is indicated in bold.

**Table 4:** Tests for cytonuclear disequilibrium.

locus	All pops	TB	SK	TUF	SHI	DOK
Cama06	0.1033	0.4591	0.8502	0.2862	0.6201	0.5016
Cama07	0.9299	0.8905	0.2927	1.0000	0.8199	0.5798
Cama08	0.1061	0.7345	0.3379	0.4637	0.0766	1.0000
Cama20	0.9222	0.2153	0.4419	0.4004	1.0000	1.0000
Cama22a	0.1333	0.4019	0.7824	0.9059	1.0000	0.5826
Cama24	0.5331	0.8498	0.3902	0.1821	0.1431	0.7850
Cmca14	0.3646	0.1264	0.6807	1.0000	0.1892	0.6086

*P* values for significance tests are shown for the Japanese population as a whole (All pops) and for individual samples: TB, Tokyo Bay site 1; SK, Shinhama-ko lagoon; TUF; Tokyo University of Fisheries; SHI, Shinhama Bay; DOK, Dokai Bay.

**Table 5:** Microsatellite diversity measures at all collection sites.

	Dokai Bay		Tokyo Bay site 1		Shinhama Bay		Shinhama-ko lagoon		Tokyo U. of Fisheries	
	*H*_S_	*A*	*H*_S_	*A*	*H*_S_	*A*	*H*_S_	*A*	*H*_S_	*A*
Cama06	0.500	2.617	0.595	2.717	0.587	2.810	0.634	2.877	0.536	2.000
Cama07	0.354	1.949	0.501	2.687	0.561	2.843	0.569	2.809	0.654	2.932
Cama08	0.768	3.851	0.744	3.935	0.725	3.859	0.691	3.610	0.731	3.618
Cama20	0.750	4.067	0.786	4.715	0.800	4.921	0.794	4.791	0.837	5.157
Cama22	0.650	4.000	0.760	4.525	0.647	3.168	0.768	4.486	0.804	4.837
Cama24	0.500	2.000	0.561	3.025	0.715	3.556	0.620	3.072	0.645	2.928
Cmca14	0.415	1.979	0.312	1.889	0.333	1.935	0.335	1.917	0.212	1.785
mean	0.562	2.923	0.608	3.356	0.624	3.299	0.630	3.366	0.631	3.322

*H*_S_, Gene diversity; *A*, allelic richness.

### Assessment of admixture

**Figure 4:**
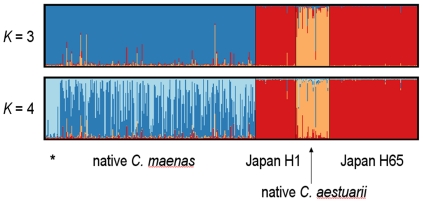
STRUCTURE clustering analysis. Each individual is represented by a vertical bar in *K* colored segments, where *K* is the number of clusters and the length of the segment is proportional to the individual's membership in the corresponding cluster. The run (out of five replicates) with the highest posterior probability is shown for *K* = 3 and *K* = 4. Black vertical bars bisecting the plots delineate pre-defined populations as indicated below the diagrams. *, separate cluster comprising native *C. maenas* samples from Iceland and the Faeroe Islands.

STRUCTURE analysis of nuclear microsatellite data without *a priori* classification of populations indicates that Japanese *Carcinus* are substantially diverged from their native congeners (Figure 4). When *K* = 3, the Japanese population forms a cluster clearly separate from the two native *Carcinus* clusters. Notably, Japanese individuals with the *C. maenas* COI haplotype (H1) are never distinguished in the analysis from those with the *C. aestuarii* haplotype (H65), even at values of *K* higher than 3. For example, at *K* = 4 population structure is observed within native *C. maenas* (individuals from Iceland and Faeroe Islands differentiated from mainland European individuals) while no structure is observed within Japan despite the presence of both *C. meanas* and *C. aestuarii* COI haplotypes (Figure 4). This result is supported by hierarchical STRUCTURE analysis of the Japanese cluster alone, which showed no sub-population structure (not shown). Assessment of likelihood values for multiple STRUCTURE runs indicates that the best supported hypothesis of true population structure occurs between *K* = 3 and *K* = 4.

**Figure 5:**
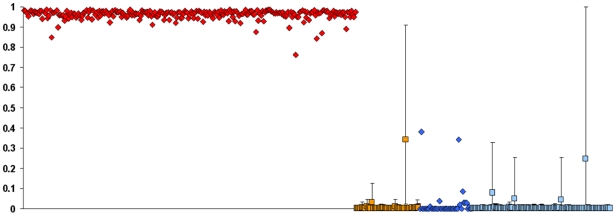
STRUCTURE analysis of admixture. Proportional coancestry (*q*_1_, shown on *y* axis) in one of two pre-defined clusters, corresponding to native *C. maenas*; coancestry in a cluster corresponding to *C. astuarii* is equal to *q*_2_ = 1−*q*_1_. Symbols are as in Figure 2: red diamonds, native *C. maenas*; blue diamonds, native *C. aestuarii*; orange squares, Japanese individuals with COI haplotype H1; light blue squares, Japanese individuals with COI haplotype H65. 95% confidence intervals are given for coancestry estimates on all Japanese individuals.

When STRUCTURE analysis was conducted with native *C. maenas* and *C. aestuarii* assigned to pre-defined populations, Japanese individuals were found to be predominantly of *C. aestuarii* ancestry (Figure 5). For all Japanese *Carcinus*, the mean coefficient of coancestry in the cluster pre-defined by native *C. aestuarii* was 0.9911. Although several individuals possessed significantly higher coefficients of coancestry in the *C. maenas* cluster, in only one case did the 95% confidence interval surrounding that coefficient fail to overlap with zero (the individual in that case possessed the *C. maenas* COI haplotype H1). Again, coancestry was completely independent of mitochondrial haplotype; mean *C. aestuarii* coancestry for Japanese individuals with haplotype H1 was 0.9881, compared to 0.9924 for individuals with haplotype H65 (*P* = 0.5309, Fisher's exact test).

## Discussion

A number of studies have previously suggested the possibility of hybridization between *C. maenas* and *C. aestuarii*. Despite estimates of divergence times between the two species on the order of 5 to 8 million years ago based on mitochondrial sequence data [32], morphometric analysis of crabs collected from Palmones, Spain at the eastern edge of the Strait of Gibraltar provides evidence of incomplete reproductive isolation [31]. Early experimental studies also reported successful laboratory crosses between *C. maenas* and *C. aestuarii* [36]. More recently, individual crabs with carapace width to length ratios typical of *C. maenas* have been observed among *C. aestuarii* populations off the coast of Tunisia in the western Mediterranean basin (Temim Deli, pers. comm.). These results lend credence to the hypothesis that natural hybrid zones may exist near the mouth of the Mediterranean Sea [28] or even further east along the North African coast, although inadequate sampling in that region currently leaves this hypothesis largely unaddressed. No *Carcinus* population in the native range has yet revealed genetic evidence of hybridization; even crabs collected from the Palmones estuary were found in a study separate from that noted above to be unambiguosly *C. maenas* by genetic criteria [29].

The introduced green crab population in Japan, however, has long been known to harbor mitochondrial DNA haplotypes from both *C. maenas* and *C. aestuarii*, and several studies have recognized the possibility of hybrid origin for this population [27], [29], [30]. The analyses presented here unambiguously support a hybrid origin for the Japanese *Carcinus* population. Phylogenetic reconstructions indicate that invasive Japanese COI haplotypes H1 and H65 derive from *C. maenas* and *C. aestuarii*, respectively (Figure 1). Genetic distances between well supported clades (mean of 10.6% Kimura 2-parameter) are consistent with a lengthy period of evolutionary independence between the two sibling species [32], reflected in the substantial genetic distance between the two invasive haplotypes (10.1%). But despite the presence of mitochondrial genomes from two species there is no evidence of significant partitioning of nuclear genetic variation by haplotype (Table 2, Figures 2 and 3), and no significant cytonuclear disequilibrium was observed in any Japanese sample, even from the more recently introduced population at Dokai Bay (Table 4).

Although the Japanese *Carcinus* population is differentiated from all sampled native populations, nuclear microsatellite data suggest a strong affinity with native *C. aestuarii* (Figure 3). Analysis of genetic admixture at microsatellite loci also indicates that Japanese *Carcinus* likely derive from introgression of the *C. maenas* mitochondrial haplotype into a predominantly *C. aestuarii* nuclear genetic background (Figure 5). This is consistent with morphological observations, which have recognized almost exclusively *C. aestuarii* morphotypes throughout Japanese populations [33], [34].

Dramatic introgression of mtDNA in the absence of substantial nuclear introgression has been observed elsewhere in a variety of taxa, including insects [37], fish [38], and mammals [39], [40], [41]. Such broad disparities in interspecific gene flow across different genomic elements have contributed to growing recognition of the semi-permeability of species boundaries and considerable speculation regarding the mechanisms driving differential introgression [42], [43]. Natural selection provides one such mechanism. For instance, genealogical analysis suggests that extremely low differentiation of mtDNA haplotypes across three species of the *Drosophila yakuba* group has been driven by post-hybridization selective displacement of mitochondrial genomes [37]. In the case of *Carcinus*, however, there is no known selective mechanism likely to drive introgression of *C. maenas* mtDNA into a *C. aestuarii* background in Japanese populations. Natural selection for particular mitochondrial types in intertidal animals has typically been associated with thermal physiology related to mitochondrial respiration [44]. However, the thermal regime of Japanese waters surrounding Honshu, Shikoku, and Kyushu Islands, where *Carcinus* has been recorded, is far more similar to conditions observed in the Mediterranean than those in Atlantic Europe [35]. This suggests that any temperature-related selection exerted on the mitochondrial genome in this region would most likely favor *C. aestuarii* types over *C. maenas*.

Alternatively, selectively neutral mechanisms may also drive mitochondrial introgression across species boundaries. Chan and Levin [45] recently demonstrated that certain models of frequency-dependent prezygotic reproductive isolation allow for very rapid biased introgression of maternally inherited genomes. This is consistent with the unidirectional hybridization hypothesis of Wirtz [46], who argued that female mate discrimination should encourage hybrid reproduction between females of a rare species and males of a common one. Both studies suggest that mitochondrial capture will be most pronounced when the maternally inherited genome is relatively rare; in other words, directional mitochondrial introgression should occur most frequently in cases where a low density population of one species interacts with a more common species, and introgression should proceed from the former into the latter. Empirical evidence for this phenomenon is widespread [41], [46]. In one dramatic example, Ferris et al. [40] observed extensive introgression of *Mus domesticus* mtDNA into *Mus musculus*, and argued that the pattern was caused by colonization of *M. musculus* territory by as few as one *M. domesticus* female. Although little is known directly of mating behavior in *C. aestuarii*, observations of *C. maenas* reveal an important role for both female choice and male competition [47], indicating that reproductive biology in this genus may satisfy the conditions of these models for selectively neutral mitochondrial introgression. The observed introgression pattern thus suggests that the hybridization event likely involved few *C. maenas* individuals introduced into a more common *C. aestuarii* population.

One remarkable aspect of the Japanese *Carcinus* population is its genetic uniformity. Introgression was observed in four populations in Tokyo Bay as well as one population in Dokai Bay, roughly 850 kilometers to the southwest (Table 1). In addition, no cytonuclear disequilibrium was observed in any of the sampled populations (Table 4). Most striking is the complete lack of significant population differentiation at nuclear loci, even between Tokyo and Dokai Bays (Table 2, Figure 4). These observations suggest one of two possible scenarios for the Japanese *Carcinus* invasion. First, genetic equilibration may have occurred subsequent to a secondary introduction of *C. maenas* individuals into an already established *C. aestuarii* population in Tokyo Bay. This hypothesis would appear to be challenged, however, by the historical record of the Japanese *Carcinus* invasion. *C. aestuarii* was first reported at a single site in Tokyo Bay in 1984 [28], [35]. Populations had been observed at several other sites throughout the bay by the end of the 1980s, and by the mid 1990s *Carcinus* was common throughout Tokyo Bay and had spread as far south as Dokai Bay [35]. Given strong evidence that the Dokai Bay population represents an expansion from the original Tokyo Bay population (see below), one would have to assume that hybridization occurred prior to the spread of *Carcinus* to Dokai Bay in the mid-1990s. The observation of both nuclear and cytonuclear equilibrium across both Tokyo and Dokai Bays thus implies rapid genetic equilibration throughout an established Tokyo Bay *C. aestuarii* population within less than ten years, between the late 1980s and mid 1990s. Given a generation time of approximately 2 years [48], this is equivalent to 5 or fewer generations between the initial hybridization event and the evolution of a panmictic introgressed population spread throughout Tokyo Bay (and perhaps more extensively throughout southern Honshu, given that the source for the Dokai Bay expansion is uncertain). This hypothesis thus would require a rather implausible confluence of events: introduction of *C. aestuarii* to Tokyo Bay followed closely by introduction of *C. maenas* to the same region, followed by extremely rapid genetic equilibration throughout the Tokyo Bay population prior to the Dokai Bay expansion.

Alternatively, it may be that the interspecific hybridization event leading to introgression of *C. maenas* mtDNA into *C. aestuarii* predates the spread of *Carcinus* in Tokyo Bay. The most parsimonious explanation for the observed genetic patterns is the anthropogenic transport of an established hybrid population from a single site in the native range of *Carcinus*, most likely from a *C. aestuarii*-dominated region in the western Mediterranean basin where Atlantic currents could occasionally introduce *C. maenas* larvae (Temim Demi and Feran Palero, pers. comm.). This would explain both the observed pattern of introgression (the past incursion of rare *C. maenas* individuals into *C. aestuarii* territory could account for biased introgression of the maternal genome) as well as the genetic uniformity of Japanese samples.

It is important to note that while some departure from that genetic uniformity has been observed, these are still consistent with this most likely invasion scenario. The only significant deviation from genetic equilibrium across the Japanese population is seen in mitochondrial haplotype frequency differences between the Tokyo and Dokai samples. These may be the result of founder effects associated with the secondary spread of *Carcinus* to Dokai Bay in the 1990's. This secondary invasion event is supported both by historical records [35] and by the genetic data presented here, which indicate that the Dokai Bay population possesses a subset of the nuclear allelic diversity present in the source population at Tokyo Bay (Table 5). Genetic drift should be more pronounced for maternally inherited haploid genomes [42], so any founder effect associated with colonization of Dokai Bay might be expected to result in greater differentiation at mitochondrial as opposed to nuclear loci. Thermal selection on the mitochondrial genome is unlikely to have driven this genetic differentiation, given similarities in temperature regime between Tokyo and Dokai Bays [35].

It should also be noted that while genetic analysis is consistent with a predominantly *C. aestuarii* origin of the nuclear genomes of Japanese green crabs, that population remains well differentiated from sampled native sources (e.g. Figure 2). It is possible that this simply reflects incomplete sampling of native *C. aestuarii*. Given that COI haplotype H65 has not been observed in the native range, it is almost certainly the case that the parental *C. aestuarii* population remains unsampled. This is not surprising; no genetic analysis has been published for *Carcinus* populations located along the North African coast, within the Mediterranean basin itself, or between Palmones, Spain (*C. maenas*) and Banyul-sur-Mer, France (*C. aestuarii*) in the northern Mediterranean, so the most likely source regions for the Japanese invasion have not been explored. Additionally, any introgression of the *C. maenas* nuclear genome into the *C. aestuarii* background could also have driven differentiation from the parental types. Unfortunately, without knowledge of the true source populations for *C. aestuarii* and *C. maenas* parental types, it is very difficult to distinguish between these two hypotheses; in fact, it is likely that some combination of the two has led to the observed differentiation from native sources. Alternatively, it is also possible that the differentiation of Japanese *Carcinus* from native samples has resulted from substantial genetic drift imposed by population bottlenecks associated with initial introduction and subsequent absence of gene flow with native sources. This phenomenon has been observed for other invasive *Carcinus* populations, but only in cases where time since introduction was greater than 100 years [29], much longer than for the Japanese invasion.

Generally speaking, a single introduction from a hybrid source appears to be the most parsimonious explanation for the Japanese invasion, and is broadly consistent with both the observed genetic pattens and known invasion history. Only one observation apparently contradicts this scenario. The *C. aestuarii* COI haplotype H65 observed in Japan belongs to a well-supported subclade of *C. aestuarii* (Figure 1), and members of that subclade have not previously been recorded outside of a sample taken from the eastern Mediterranean (Naples, Italy) [29]. However, given the aforementioned problems with existing *C. aestuarii* sampling, the strength of this evidence against the proposed invasion scenario is limited. The hypothesis supported here would predict that the subclade in question is much more widely distributed than previously observed; specifically, haplotype H65 (and, presumably, related haplotypes) is expected to have a native range extending well into the western Mediterranean.

The results of genetic analyses presented here may prove relevant to assessments of future risks posed by *Carcinus* in Japan and surrounding locales. For instance, a recently developed model of green crab range expansion in the region suggests that primary introduction from the native range is likely a rare event [35], consistent with the most likely invasion scenario detailed above. Given the proliferation of recent studies illustrating the potential risks posed by multiple introductions [12], [14], this would appear to be a welcome finding. However, one of the concerns associated with multiple introductions is the admixture of previously allopatric evolutionary lineages resulting in novel genetic complexes with unexpected and potentially highly invasive phenotypes [16], [17], [18]. The emergence of such genetic novelty frequently has been cited as an important factor in determining the invasiveness of hybrid populations, particularly among plant taxa [6], [13], [15]. In the case of Japanese *Carcinus*, it appears that such admixture may in fact predate the invasion. This raises important questions regarding the possibility of ecologically relevant distinctions between *C. maenas*, *C. aestuarii*, and their hybrids. The ecology of *C. maenas* has been particularly well studied, and organismal and ecological traits likely to affect range expansion and invasiveness have been incorporated into various risk assessments [49], [50], [51], [52]. Comparatively little is known regarding the ecology of *C. aestuarii*, and what is known derives largely from study of Japanese populations [34] and observations of abiotic characteristics of the recorded native range (e.g. seawater temperatures [35]). However, the genetic analyses presented here recommend some caution in assuming that Japanese *Carcinus* will reflect the ecological characteristics of either parent species. For instance, introgression of *C. maenas* mitochondrial genomes throughout the Japanese population suggests the possibility that those populations possess capacity for thermal adaptation significantly different from native *C. aestuarii*. Invasion of Hokkaido Island, with minimum seawater temperatures apparently more suited to *C. maenas* than *C. aestuarii*, therefore may be more likely than assumed by current risk assessments [35]. The analysis presented here thus provides further evidence for an important role of genetic analysis in better understanding evolutionary history potentially relevant to the effective management of invasive populations [23].

## Materials and Methods

### Sample collection and processing

**Figure 6:**
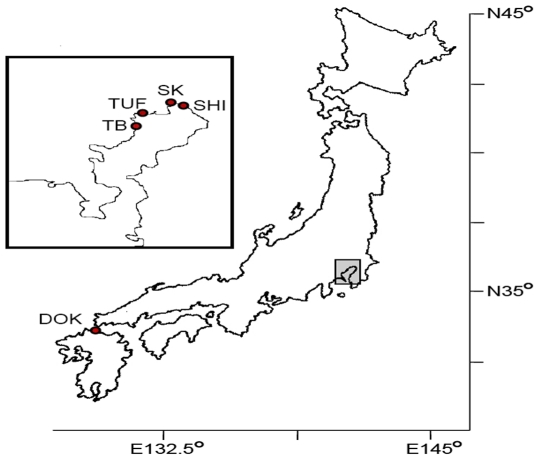
Map showing distribution of collection sites. Tokyo Bay (gray rectangle) is shown inset. Site IDs are as in Table 1.

Live crabs were collected from four sites in Tokyo Bay in 1995 and 1996 and a single site in Dokai Bay in 1997 (Table 1, Figure 6). Specimens were frozen at −20°C or preserved in 70–95% ethanol for DNA extraction, and DNA was extracted from frozen or preserved gill tissue using the protocol of Geller et al. (1997). Prior to PCR amplification, all DNA samples were further purified using DNeasy Tissue Kits (QIAGEN). All genetic data used in the current study, including data from native *C. maenas* and *C. aestuarii*, have been described previously [29].

### Molecular methods

PCR amplification of the mitochondrial cytochrome *C* oxidase subunit I (COI) was conducted as previously described using universal primers LCO1490 (GGTCAACAAATCATAAAGATATTGG) *and HCO2198 (TAAACTTCAGGGTGACCAAAAAATCA) for C. maenas haplotypes and degenerate universal primers COIF-PR115 (TCWACNAAYCAYAARGAYATTGG) and COIR-PR114 (ACYTCNGGRTGNCCRAARARYCA) for C. aestuarii [29]. PCR products were sequenced directly in both forward and reverse directions using amplification primers. All sequence editing was done in Sequencher v4.6. Carcinus microsatellite loci Cma01EPA, Cma02EPA, Cma03EPA, Cma04EPA, Cma05EPA, Cma08EPA, Cma09EPA, Cma14EPA, and Cma16EPA were amplified using previously described cycling parameters [29], [53]. Microsatellites were scored using GeneMarker software (v 1.5).*

### Phylogenetic analysis

COI sequences were aligned using ClustalX [54] and trimmed to the length of the shortest sequence, resulting in 443 bp of unambiguously aligned, gap-less sequence for phylogenetic analysis. All known *Carcinus* COI haplotypes were included in the analysis [29], [32]. Mean Kimura 2-parameter genetic distances between *C. maenas* and *C. aestuarii* were calculated in MEGA v4.0 [55]. Phylogenetic relationships were determined by Bayesian inference using MrBayes v. 3.1.2 [56]. Analysis was performed assuming a Generalized Time Reversible model with gamma distribution of substitution rates and a proportion of invariant sites (GTR+I+G), as recommended by the software MODELTEST [57]. The search was run with four chains for 10^6^ generations, with sampling every 100 generations and 2,500 trees discarded as burnin. Trees were rooted using sequences from three outgroup species belonging to the family Portunidae, *Callinectes sapidus* (GenBank accession #AY682079), *Charybdis japonica* (#EU586120), and *Portunus sanguinolentus* (#EU284152). Kimura 2-parameter genetic distances between invasive haplotypes and within and between *C. maenas* and *C. aestuarii* were calculated in MEGA v. 4 [55].

### Analysis of population genetic structure

Genotypic data were assessed for departures from Hardy-Weinberg equilibrium (HWE) using Fisher's exact test in GENEPOP v3.4 [58]. Locus by locus cytonuclear disequilibrium was assessed using the software CNDWin [59], with 10,000 Markov Chain Monte Carlo repetitions. Genetic structure was determined by conducting analysis of molecular variance (AMOVA) and pairwise analysis of population differentiation (*F*_ST_) on microsatellite data with ARLEQUIN v. 3.0 [60]; statistical significance was assessed with 1000 permutations. For AMOVA, samples were grouped either by collection region (Tokyo Bay vs. Dokai Bay) or by mitochondrial haplotype (*C. maenas* vs. *C. aestuarii*) and tested for partitioning of genetic variance within and between groups. Significance of difference in the distribution of the two *Carcinus* COI haplotypes between Tokyo and Dokai Bays was determined by Fisher's exact test. In addition, genetic relationships between individual multi-locus genotypes were assessed using Factorial Correspondence Analysis (FCA) conducted with the software GENETIX v4.05.2 [61]. Allele frequency distributions were determined using MSANALYZER v. 4.0 [62], and gene diversity and allelic richness were calculated in FSTAT v. 2.9.3.2 [63]. To assess relationships between Japanese and native *Carcinus* populations, pairwise Cavalli Sforza-Edwards chord distances were calculated based on microsatellite data using MICROSATELLITE ANALYZER [62], with 1000 bootstrap replicates to assess statistical support. Relatedness trees were constructed based on chord distances using the neighbor joining algorithm, and a majority rule bootstrap consensus tree was built using the programs NEIGHBOR and CONSENSE in PHYLIP v. 3.65 [64]. Japanese individuals were grouped according to COI haplotype for this analysis.

### Assessment of admixture

To assess admixture in the Japanese *Carcinus* population, Bayesian model-based cluster analysis was implemented using the program STRUCTURE v.2.2 [65], which assigns individual genotypes to populations based on minimization of both Hardy Weinberg and linkage disequilibrium within those populations. Two different tests were conducted using this approach. Initially, the program was allowed to assign individuals to clusters without *a priori* classification of populations. Known *C. maenas* and *C. aestuarii* individuals from the native range of both species, as well as all Japanese *Carcinus*, were assigned probabilistically to populations or jointly to multiple populations if their genotypes indicated admixture. For this analysis, likelihood of models was assessed with *K* (the user-defined number of clusters) ranging between 1 and 5. In addition, the coancestry of Japanese individuals in parental gene pools defined by native *C. maenas* and native *C. aestuarii* was determined by adopting an ancestry model that used prior population information to determine clustering. Specifically, native *C. maenas* and *C. aestuarii* individuals were classified as known samples belonging to two pre-defined parental clusters, and all Japanese individuals were classified as of unknown origin. By setting *K* = 2, this procedure allowed estimation of admixture proportions of known *C. maenas* and *C. aestuarii* allelic states in the test population (Japanese *Carcinus*). For both analyses, five independent runs were conducted, each run consisting of 1,000,000 iterations with the first 100,000 iterations discarded as burn-in. STRUCTURE results were visualized using the software DISTRUCT v. 1.1 [66].

## References

[1] Lee CE (2002). Evolutionary genetics of invasive species.. Trends in Ecology & Evolution.

[2] Vellend M, Harmon LJ, Lockwood JL, Mayfield MM, Hughes AR (2007). Effects of exotic species on evolutionary
diversification.. Trends in Ecology & Evolution.

[3] Wares JP, Hughes AR, Grosberg RK, Sax DF, Stachowicz JJ, Gaines SD (2005). Mechanisms that drive evolutionary change: insights from species
introductions and invasions.. Species Invasions: Insights into Ecology, Evolution, and
Biogeography.

[4] Ellstrand NC, Schierenbeck KA (2000). Hybridization as a stimulus for the evolution of invasiveness in
plants?. Proceedings of the National Academy of Sciences of the United States of
America.

[5] Hanfling B (2007). Understanding the establishment success of non-indigenous fishes:
lessons from population genetics.. Journal of Fish Biology.

[6] Mallet J (2007). Hybrid speciation.. Nature.

[7] Wissemann V (2007). Plant evolution by means of hybridization.. Systematics and Biodiversity.

[8] Largiader C, Nentwig W (2007). Hybridization and introgression between native and alien
species.. Biological Invasions.

[9] Levin DA, FranciscoOrtega J, Jansen RK (1996). Hybridization and the extinction of rare plant
species.. Conservation Biology.

[10] Rhymer JM, Simberloff D (1996). Extinction by hybridization and introgression.. Annual Review of Ecology and Systematics.

[11] Vila M, Weber E, D'Antonio C (2000). Conservation implications of invasion by plant
hybridization.. Biological Invasions.

[12] Dlugosch KM, Parker IM (2008). Founding events in species invasions: genetic variation, adaptive
evolution, and the role of multiple introductions.. Molecular Ecology.

[13] Prentis PJ, Wilson JRU, Dormontt EE, Richardson DM, Lowe AJ (2008). Adaptive evolution in invasive species.. Trends in Plant Science.

[14] Roman J, Darling JA (2007). Paradox lost: genetic diversity and the success of aquatic
invasions.. Trends in Ecology & Evolution.

[15] Rieseberg LH, Kim SC, Randell RA, Whitney KD, Gross BL (2007). Hybridization and the colonization of novel habitats by annual
sunflowers.. Genetica.

[16] Facon B, Pointier JP, Jarne P, Sarda V, David P (2008). High genetic variance in life-history strategies within invasive
populations by way of multiple introductions.. Current Biology.

[17] Kolbe JJ, Larson A, Losos JB (2007). Differential admixture shapes morphological variation among
invasive populations of the lizard *Anolis
sagrei*.. Molecular Ecology.

[18] Lavergne S, Molofsky J (2007). Increased genetic variation and evolutionary potential drive the
success of an invasive grass.. Proceedings of the National Academy of Sciences of the United States of
America.

[19] Hall RJ, Ayres DR (2009). What can mathematical modeling tell us about hybrid
invasions?. Biological Invasions.

[20] Kolbe JJ, Glor RE, Schettino LRG, Lara AC, Larson A (2007). Multiple sources, admixture, and genetic variation in introduced
*Anolis* lizard populations.. Conservation Biology.

[21] Holsbeek G, Mergeay J, Hotz H, Plotner J, Volckaert FAM (2008). A cryptic invasion within an invasion and widespread
introgression in the European water frog complex: consequences of
uncontrolled commercial trade and weak international
legislation.. Molecular Ecology.

[22] Hufbauer RA, Sforza R (2008). Multiple introductions of two invasive *Centaurea*
taxa inferred from cpDNA haplotypes.. Diversity and Distributions.

[23] Geller JB, Darling JA, Carlton JT (2010). Genetic perspectives on marine biological
invasions.. Annual Review of Marine Science.

[24] Bastrop R, Jurss K, Sturmbauer C (1998). Cryptic species in a marine polychaete and their independent
introduction from North America to Europe.. Molecular Biology and Evolution.

[25] Davidson SK, Haygood MG (1999). Identification of sibling species of the bryozoan *Bugula
neritina* that produce different anticancer bryostatins and
harbor distinct strains of the bacterial symbiont *Candidatus
endobugula sertula*.. Biological Bulletin.

[26] Folino-Rorem NC, Darling JA, D'Ausilio CA (2009). Genetic analysis reveals multiple cryptic invasive species of the
hydrozoan genus *Cordylophora*.. Biological Invasions.

[27] Bagley MJ, Geller JB, Pederson J (2000). Microsatellite analysis of native and invading populations of
European green crabs.. Marine Bioinvasions: proceedings of the first national
conference.

[28] Carlton JT, Cohen AN (2003). Episodic global dispersal in shallow water marine organisms: the
case history of the European shore crabs *Carcinus maenas*
and *C. aestuarii*.. Journal of Biogeography.

[29] Darling JA, Bagley MJ, Roman J, Tepolt CK, Geller JB (2008). Genetic patterns across multiple introductions of the globally
invasive crab genus *Carcinus*.. Molecular Ecology.

[30] Geller JB, Walton ED, Grosholz ED, Ruiz GM (1997). Cryptic invasions of the crab *Carcinus* detected
by molecular phylogeography.. Molecular Ecology.

[31] Clark PF, Neale M, Rainbow PS (2001). A morphometric analysis of regional variation in
*Carcinus* Leach, 1814 (Brachyura: Portunidae:
Carcininae) with particular reference to the status of the two species
*C. maenas* (Linnaeus, 1758) and *C.
aestuarii* Nardo, 1847.. Journal of Crustacean Biology.

[32] Roman J, Palumbi SR (2004). A global invader at home: population structure of the green crab,
*Carcinus maenas*, in Europe.. Molecular Ecology.

[33] Yamada SB, Hauck L (2001). Field identification of the European green crab species:
*Carcinus maenas* and *Carcinus
aestuarii*.. Journal of Shellfish Research.

[34] Chen RB, Watanabe S, Yokota M (2004). Feeding habits of an exotic species, the Mediterranean green crab
*Carcinus aestuarii*, in Tokyo Bay.. Fisheries Science.

[35] Koike F, Iwasaki K (2011). A simple range expansion model of multiple pathways: the case of
nonindigenous green crab *Carcinus aestuarii* in Japanese
waters.. Biological Invasions.

[36] Demeusy N, Veillet A (1953). Sur l'existence de deux populations de *Carcinus
maenas* Pennant et sur les caractères morphologiques qui
les distinguent.. Compte Rendu Hebdomadaire des Séances de l'Académie
des Sciences, Paris.

[37] Bachtrog D, Thornton K, Clark A, Andolfatto P (2006). Extensive introgression of mitochondrial DNA relative to nuclear
genes in the *Drosophila yakuba* species
group.. Evolution.

[38] Bernatchez L, Glemet H, Wilson CC, Danzmann RG (1995). Introgression and fixation of arctic char (*Salvelinus
alpinus*) mitochondrial genome in an allopatric population of
brook trout (*Salvelinus fontinalis*).. Canadian Journal of Fisheries and Aquatic Sciences.

[39] Berthier P, Excoffier L, Ruedi M (2006). Recurrent replacement of mtDNA and cryptic hybridization between
two sibling bat species *Myotis myotis* and *Myotis
blythii*.. Proceedings of the Royal Society B-Biological Sciences.

[40] Ferris SD, Sage RD, Huang CM, Nielsen JT, Ritte U (1983). Flow of mitochondrial DNA across a species
boundary.. Proceedings of the National Academy of Sciences of the United States of
America-Biological Sciences.

[41] Good JM, Hird S, Reid N, Demboski JR, Steppan SJ (2008). Ancient hybridization and mitochondrial capture between two
species of chipmunks.. Molecular Ecology.

[42] Ballard JWO, Whitlock MC (2004). The incomplete natural history of mitochondria.. Molecular Ecology.

[43] Wu CI (2001). The genic view of the process of speciation.. Journal of Evolutionary Biology.

[44] Somero GN (2002). Thermal physiology and vertical zonation of intertidal animals:
Optima, limits, and costs of living.. Integrative and Comparative Biology.

[45] Chan KMA, Levin SA (2005). Leaky prezygotic isolation and porous genomes: Rapid
introgression of maternally inherited DNA.. Evolution.

[46] Wirtz P (1999). Mother species-father species: Unidirectional hybridization in
animals with female choice.. Animal Behaviour.

[47] Berrill M, Arsenault M (1982). Mating behavior of the green shore crab *Carcinus
maenas*.. Bulletin of Marine Science.

[48] Yamada SB (2000). Global Invader: The European Green Crab.

[49] See KE, Feist BE (2010). Reconstructing the range expansion and subsequent invasion of
introduced European green crab along the west coast of the United
States.. Biological Invasions.

[50] Compton TJ, Leathwick JR, Inglis GJ (2010). Thermogeography predicts the potential global range of the
invasive European green crab (*Carcinus
maenas*).. Diversity and Distributions.

[51] Derivera CE, Hitchcock NG, Teck SJ, Steves BP, Hines AH (2007). Larval development rate predicts range expansion of an introduced
crab.. Marine Biology.

[52] deRivera CE, Ruiz GM, Hines AH, Jivoff P (2005). Biotic resistance to invasion: Native predator limits abundance
and distribution of an introduced crab.. Ecology.

[53] Tepolt CK, Bagley MJ, Geller JB, Blum MJ (2006). Characterization of microsatellite loci in the European green
crab (*Carcinus maenas*).. Molecular Ecology Notes.

[54] Thompson JD, Gibson TJ, Plewniak F, Jeanmougin F, Higgins DG (1997). The CLUSTAL_X windows interface: Flexible strategies for multiple
sequence alignment aided by quality analysis tools.. Nucleic Acids Research.

[55] Tamura K, Dudley J, Nei M, Kumar S (2007). MEGA4: Molecular evolutionary genetics analysis (MEGA) software
version 4.0.. Molecular Biology and Evolution.

[56] Ronquist F, Huelsenbeck JP (2003). MrBayes 3: Bayesian phylogenetic inference under mixed
models.. Bioinformatics.

[57] Posada D, Crandall K (1998). Modeltest: Testing the model of DNA substitution.. Bioinformatics.

[58] Raymond M, Rousset F (2004).

[59] Basten CJ, Asmussen MA (1997). The exact test for cytonuclear disequilibria.. Genetics.

[60] Excoffier L, Laval G, Schneider S (2005). Arlequin version 3.0: An integrated software package for
population genetics data analysis.. Evolutionary Bioinformatics.

[61] Belkhir K, Borsa P, Chikhi L, Raufaste N, Bonhomme F (2004). GENETIX 4.05, logiciel sous Windows TM pour la génétique
des populations.

[62] Dieringer D, Schlötterer C (2002). Microsatellite analyser (MSA): A platform independent analysis
tool for large microsatellite data sets.. Molecular Ecology Notes.

[63] Goudet J (2001).

[64] Felsenstein J (1989). PHYLIP: Phylogeny inference package (version
3.2).. Cladistics.

[65] Falush D, Stephens M, Pritchard JK (2003). Inference of population structure using multilocus genotype data:
Linked loci and correlated allele frequencies.. Genetics.

[66] Rosenberg NA (2004). DISTRUCT: A program for the graphical display of population
structure.. Molecular Ecology Notes.

